# Adult-onset demyelinating neuropathy associated with FBLN5 gene mutation

**DOI:** 10.5414/NP301011

**Published:** 2017-03-23

**Authors:** Si Cheng, He Lv, Wei Zhang, Zhaoxia Wang, Xin Shi, Wei Liang, Yun Yuan

**Affiliations:** Department of Neurology, Peking University First Hospital, Beijing, China; *These authors contributed equally to the manuscript.

**Keywords:** FBLN5, Charcot-Marie-Tooth disease, demyelinating neuropathy, nerve ultrasound

## Abstract

Rare forms of autosomal-dominant Charcot-Marie-Tooth disease (AD-CMT) may be associated with mutations in Fibulin-5 (FBLN5) as AD-CMT is genetically heterogeneous. Here, we report the first pathological study of an Asian family. The proband was a 46-year-old man with slowly progressive distal numbness and weakness for 12 years. He had a history of diabetes mellitus for 12 years. His mother was 81 years old and had mild polyneuropathy. His 16-year-old daughter was asymptomatic. The nerve conduction velocities (NCVs) and compound muscular action potential (CMAP) amplitudes were moderately to severely reduced in the proband, and moderately reduced in his daughter and mother. A sensory response could not be elicited in the proband and was moderately to severely decreased in the daughter and mother. Nerve ultrasound indicated a general enlargement of the peripheral nerves in the proband, daughter, and mother. A sural nerve biopsy from the proband demonstrated a pronounced depletion of myelinated fibers, thin myelinated fibers, and onion-bulb formations. A reported heterozygous mutation of c.1117C>T in FBLN5 was identified in the proband, mother, and daughter. These findings confirm a novel subtype of AD-CMT 1 due to a mutation in the FBLN5 gene.

## Introduction 

Fibulin-5 (FBLN5), an extracellular matrix calcium-binding glycoprotein, is essential for elastic fiber formation [1]. FBLN5 mutations are associated with three distinct human diseases, including age-related macular degeneration, cutis laxa, and Charcot-Marie-Tooth disease (CMT) [2]. The mutation of c.1117C>T (p.R373C) of the FBLN5 gene has been reported to cause AD-CMT1 in two unrelated families from Austria [2] and the Czech Republic [3] in a European population. The onset of the disease was in adulthood. The main clinical features included symmetric distal muscle atrophy, weakness and numbness in all limbs, which initially involved the upper limbs described in the Austrian patients [2]. The Austrian family also exhibited age-related macular degeneration. The slowing of nerve conduction velocities (NCVs) presented at the early stage of the disease, even in asymptomatic carriers, which indicated demyelinating neuropathy [2]. However, there is no morphological description of the peripheral nerves. Here, we report the first Chinese family with c.1117C>T mutation of FBLN5. We performed nerve ultrasounds in the family and a pathological examination of the sural nerve in the proband. 

## Materials and methods 

### Clinical data 

**Figure 1.**
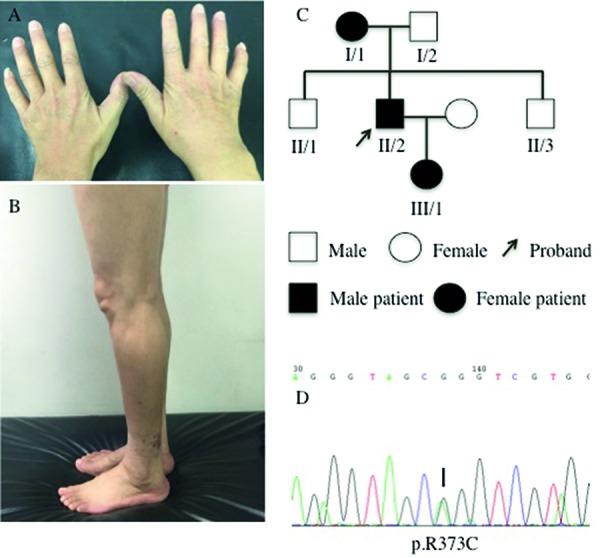
A: Atrophies of hand muscles in the proband. B: Calf atrophy in the proband (II/2). C: Pedigree of the family. D: The c.1117C>T mutation in the FBLN5 gene, which caused arginine to be substituted by cysteine at nucleotide 373.

The proband was a 46-year-old man. At 34 years of age, he began to feel numbness and weakness of both hands and was experiencing difficulty with buttons. Diabetes mellitus (DM) was simultaneously diagnosed due to an increased blood sugar concentration. At the age of 36, he complained of gait difficulty with frequent tripping, which was accompanied by sensory disturbances of both lower legs. He presented with adiaphoresis and was insensitive to temperature and touch in his hands and feet. A neurologic examination indicated that the sensitivities to pinprick and touch were reduced in both hands and below the knees. The sensitivity to vibration in both lower limbs was decreased. The muscle strength was 4/5 on finger extension (Medical Research Council Scale, grades 0 – 5), 4/5 on hand abduction and adduction, 4/5 on foot plantar flexion, and 3/5 on foot dorsiflexion bilaterally. The deep tendon reflexes were decreased. Steppage gait and atrophy of the interosseous, thenar, hypothenar, and intrinsic feet and calf muscles were noted. The Charcot-Marie-Tooth disease neuropathy score second version (CMTNS) was 20. Chronic inflammatory demyelinating polyradiculoneuropathy was clinically diagnosed because of an increased protein level in the cerebrospinal fluid and electrophysiological evidence of demyelinating neuropathy. However, the clinical symptoms did not significantly improve after intravenous immuneoglobulin. In the next 10 years, the weakness of the distal muscles slowly deteriorated in the legs. At 46 years old, the neurological examination indicated that the sensory loss region did not enlarge in both the hands and feet. The muscle strength of the upper limb did not decrease. The strength of the feet dorsiflexion decreased to 1/5. Steppage gait and muscle atrophy in the hand (Figure 1A) and calf (Figure 1B) were not significantly developed. Deep tendon reflexes were absent. The CMTNS was 21. He had a normal visual acuity and cutaneous situation. An increased glycated hemoglobin A1c of 9.10% (typically < 6.0%) was noted. Serum creatine kinase, autoimmune antibodies, folic acid, and vitamin B12 were within normal limits. 

In this family pedigree (Figure 1C), his 81-year-old mother (I/1) exhibited mild peripheral neuropathy with numbness in both the hands and feet and a mild decrease in visual acuity. Physical examination revealed pes cavus, macular degeneration, and cataract of the right eye. The sensitivities to pinprick, touch, and temperature were decreased below the bilateral elbows and ankle joints. The sensitivity to vibration of both lower limbs was significantly decreased. The muscle strength was 4/5 on finger extension and normal in the other muscle groups of all limbs. No muscle atrophy was identified. Deep tendon reflexes were absent. Ophthalmological examination revealed opacity in the lens of right eye and subretinal fibrosis caused by a choroidal neovascularization due to age-related macular degeneration in the left eye. The proband’s 16-year-old daughter (III/1) had no clinical symptoms of peripheral neuropathy. Physical examination indicated normal sensory and muscle strength in all limbs. Deep tendon reflexes were absent. The proband’s 43-year-old brother did not exhibit clinical symptoms. Physical examination indicated no abnormalities. 

## Methods 

The Ethics Committee of Peking University First Hospital, China, approved this study. Written informed consents were signed by the family members prior to initiation of the investigation in accordance with the Declaration of Helsinki. 

### Electrophysiological examinations 

Electrophysiological examinations were performed in the proband and at-risk family members. The NCVs were recorded with surface stimulating and recording electrodes. The compound muscle action potentials (CMAPs) of the median and ulnar nerves were recorded from the abductor pollicis brevis and the abductor digiti quinti, respectively, with stimulation at the wrist and elbow. The CMAP of the peroneal nerve was recorded from the extensor digitorum brevis with stimulation at the ankle and knee. The distal motor latency (DML), motor nerve conduction velocities (MNCVs), and distal CMAP amplitude were measured. The sensory nerve action potentials (SNAPs) of the median and ulnar nerves were obtained at the wrist following stimulation of the second and fifth digits, respectively. The SNAP of the sural nerve was antidromically recorded at the ankle following stimulation of the calf. The SNAP amplitudes and sensory nerve conduction velocity (SNCV) were measured in the peroneal nerve. 

### Nerve ultrasonography 

Ultrasonography was performed in the proband, daughter, and mother using a Philips ultrasound system (iU Elite, Bothell, WA, USA). A 17-MHz linear array transducer for the superficial nerves was used. The nerve size, echogenicity, and vascularization were evaluated. All nerves were measured on transverse sections with determination of the cross-sectional area (CSA) within the hyperechoic rim that surrounded the nerve using the continuous tracing technique. The peripheral nerves were measured bilaterally at the following sites, which represent the same sites employed in a previous study [4]: (1) median nerve at the distal wrist crease, distal forearm, proximal forearm, antecubital fossa and mid-humerus; and (2) ulnar nerve at the distal wrist crease, distal forearm, arterial split, and tip of the medial epicondyle. The reference values were obtained from a previous study of healthy adults [4]. 

### Histopathological studies 

A sural nerve biopsy was performed in the proband. One piece of the nerve was fixed in 4% formaldehyde, paraffin-embedded, cut into 8-µm sections, and stained with hematoxylin and eosin, Luxol Fast Blue, and Congo Red. The remaining specimen was fixed in 3% glutaraldehyde, post-fixed in 1% osmium tetroxide, dehydrated through serial alcohol baths, and embedded in Epon 812. Semi-thin sections for light microscopy were stained with toluidine blue. Ultrathin sections were contrasted with uranyl acetate and lead citrate and subsequently examined via electron microscopy. Images of randomly selected nerve areas were measured using the NIS-Elements BR 3.2 program. 

### Genetic test 

Genomic DNA was extracted from peripheral blood samples from all four family members. The genetic test was performed in the proband using targeted next-generation sequencing, which was conducted on selected subjects using an Agilent SureDesign Panel kit (0.4-Mb, for neuromuscular disorders) (Agilent, Santa Clara, CA, USA) and an Illumina HiSeq 2500 sequencer (Illumina, San Diego, CA, USA). The reads were aligned for single-nucleotide polymorphism (SNP) calling and subsequent analysis. Nonsynonymous/splice acceptor and donor site/insertion or deletion (NS/SS/InDel) variants in the dbSNP v137, ESP6500, and 1000 genome were removed. Synonymous changes were filtered using SIFT software (http://sift.jcvi.org). PMP22 duplication was excluded by multiplex ligation-dependent probe amplification (MLPA). Sanger sequencing with specific primers was conducted to confirm the FBLN5 mutation of the proband (II/2) and his family members (I/1, III/1, and II/3). 

## Results 

### Electrophysiological studies 

The results of the electrophysiological examination are provided in Table 1. The MNCVs of the median and ulnar nerves were moderately to severely reduced in the proband and moderately reduced in his daughter and mother. The CMAP amplitudes in the upper limbs were severely reduced in the proband, with no abnormalities in the daughter and mother. The lowest MNCV of the peroneal and tibial nerves was identified in the proband who also had substantially reduced CMAP amplitudes of these nerves. DML was delayed in the three patients. A sensory response could not be elicited in the lower limbs of the proband. SNAPs were obtained in the daughter and mother with a normal amplitude. An F wave and H reflex could not be elicited in the proband. The F wave conduction velocities were moderately reduced, and the latency to an F wave was moderately prolonged in the peroneal nerve in the daughter and mother. These findings were consistent with a mixed axonal-demyelinating neuropathy in the proband and demyelinating neuropathy in the daughter and mother. 

### Nerve ultrasound 

**Figure 2.**
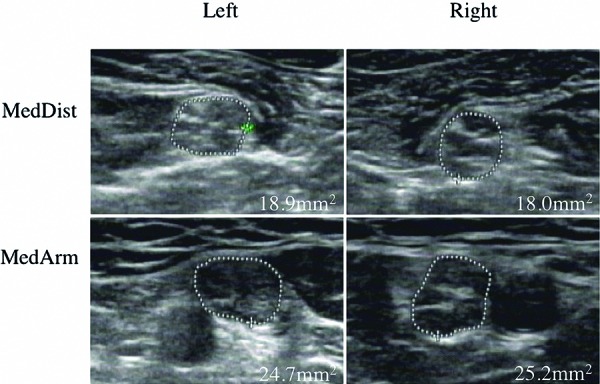
Enlargement of peripheral nerves in the proband. (MedDist = distal forearm along the median nerve; MedArm = mid-humerus along the median nerve).

The proband, daughter, and mother exhibited a general increment of the CSAs. The CSAs of the bilateral median nerves were 18.76 ± 4.15 mm^2^ in the proband, 16.46 ± 4.15 mm^2^ in the daughter, and 21.82 ± 6.73 mm^2^ in the mother. All values were significantly larger than the normal reference size (5.6 – 9.1 mm^2^). The CSAs of the bilateral ulnar nerves were 16.33 ± 4.22 mm^2^ in the proband, 12.44 ± 2.51 mm^2^ in the daughter, and 14.35 ± 3.81 mm^2^ in the mother. All data were substantially increased compared with the normal reference size (4.1 – 6.7 mm^2^). A high hypo-echoic fraction was noted in multiple examination sites. The fascicle size was larger compared with healthy individuals in the multifocal of several nerves and was not restricted to the carpal tunnel and sulcus. Both increased CSA and hypo-echogenicity of the median and ulnar nerves were more notable in the upper arm compared with the forearm (Figure 2). No increased vascularization was identified. 

### Nerve biopsy 

**Figure 3.**
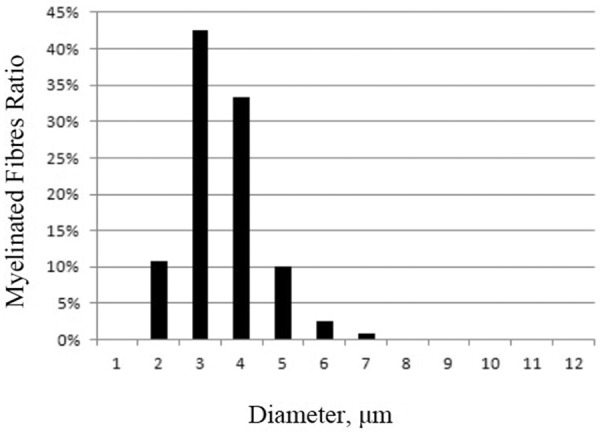
Diameter distribution of myelinated fibers in the proband. There is a substantial loss of large myelinated fibers, with a shift towards smaller diameters.

**Figure 4.**
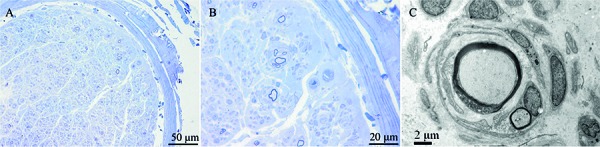
A, B: Severe loss of myelinated fibers and onion-bulb formations (OBFs). C: An onion bulb formation surrounding a cluster of two regenerating/remyelinating fibers.

The total transverse fascicular area was between 0.23 and 0.61 mm^2^. The density of myelinated fibers (MFs) was 605 ± 160/mm^2^ (7,400 ± 1,300/mm^2^ in normal subjects). The densities of both the large and small MFs were decreased, with a predominant loss of myelinated fibers with large diameters (Figure 3). One predominant change included the presence of onion bulb formations (OBFs) that involved almost all remaining MFs (Figure 4A, B). OBFs have 0 or 1 thin myelinated axon. The ratio of the diameter of axon to axon with myelin (G-ratio) was 0.72 – 0.86 (typically 0.5 – 0.7 [5]). Large axons without myelin were occasionally identified. There were a few axonal sprouts. Wallerian degeneration and lymphocyte infiltration were not identified. Several capillaries exhibited thickening of the basement membrane. 

Electron microscopic observation confirmed that the OBFs consisted of 2 – 5 concentric layers of Schwann cell processes. The individual axon was denuded or had thin myelin relative to the axon (Figure 4C). The unmyelinated fibers were frequently incorporated into the circumferential lamellae of the OBFs. Occasionally, chromatin condensation of Schwann cells was identified, which suggested apoptosis. Collagen pockets were diffusely distributed. Endoneurial fibroblast proliferation was well documented. 

### Genetic test 

**Figure 5.**
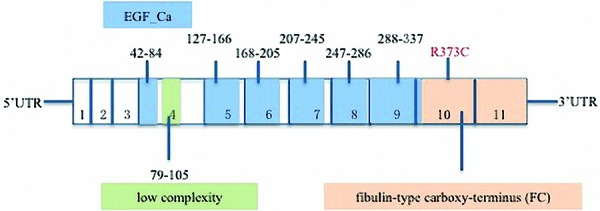
Schematic model of FBLN5 indicates epidermal growth factor calcium-binding domains (EGF_Ca) in blue, the low complexity domain in green (domain boundaries are indicated with amino acid positions above the exons), and the FBLN-type carboxy-terminus (FC) in yellow; exons 1 – 11 and disease-associated mutations. Mutated FBLN5 is indicated in red color.

A previously reported heterozygous mutation, c.1117C>T, was detected in exon 10 of the FBLN5 gene (Figure 5), with arginine substituted with cysteine at nucleotide 373 (p.R373C) (Figure 1D). The same mutation was identified in the patient’s mother and daughter; however, it was absent in the healthy brother. 

## Discussion 

The present family with AD-CMT1 caused by an FBLN5 gene mutation is the third family worldwide and the first Asian report outside of a European population [3, 4]. 

We further confirmed that the clinical onset of the disease occurred in adulthood or at an elderly age [3, 4]. Patients less than 30 years old may be asymptomatic [4]. However, a detailed neurological examination indicated signs of peripheral neuropathy, even in the second decade in the proband’s daughter. The main clinical features indicated a motor-sensory neuropathy not only in our symptomatic patients but also in other reports [3, 4]. In the reported families, the weakness developed from the distal legs in most patients. A stocking-like sensory loss appeared in old affected patients. A Carpal tunnel syndrome-like feature was exhibited in our proband and in patients in other reports [2]. 

Our study further confirmed that senior macular degeneration is a common symptom. In the 81-year-old mother of the proband, an optic examination indicated macular degeneration. Age-related macular degeneration was identified in one Austrian patient at age 81 [2], in contrast to no individuals in the Czech family in which the oldest patient was 61 years old [3]. Hyperelastic skin was described only in patients from the Austrian family [2] and not in the Czech family [3] or the present family. Diabetes mellitus in the proband is a coincident disease, similar to CMT1A [6]. 

The ultrasound examination in the affected member of this family indicated a general hypertrophic neuropathy, which helped to classify as CMT1. The CSAs of the peripheral nerves in the upper limbs were consistently increased 2- to 3-times compared with the normal reference size. Our finding further confirmed that generalized and homogeneous nerve enlargement is a common feature for CMT1 because the same changes also appeared in CMT1A [7, 8]. This feature is different from chronic inflammatory demyelinating polyneuropathy, which exhibited a segmental enlargement of the peripheral nerves [7]. 

Electrophysiology in this family helped to classify as CMT1 and similar changes presented in the other reported cases of FBLN5-related AD-CMT1 disease [2, 3] and CMT1A [9]. We further confirmed that slowing of the MNCVs appeared in asymptomatic carriers [2]. The severely deceased amplitude of the CMAP in the proband may be a result of the coincidence of diabetes mellitus. Diabetes has been associated with more severe motor and sensory impairments with lower amplitudes of the CMAP and SNAPs in patients with CMT1A [6]. The CMAP amplitudes in the daughter and mother did not exhibit abnormalities because they did not have diabetes mellitus. 

A sural nerve biopsy in the proband was the first to find severe demyelinating neuropathy for FBLN5-related AD-CMT1 disease, which was characterized by the loss of myelinated fibers and numerous OBFs with uniformly thin myelin sheaths. Both light and electron microscopic observations indicated a high G-ratio for the remaining myelinated fibers, which have typically been described as hypomyelination in CMT1A or CMT1E [10, 11]. A thickened basement membrane of the capillary in the proband is a common vascular pathology of diabetes mellitus. Based on myelinated fibres with a high G-ratio, it is impossible that secondary changes are due to axonal degeneration in diabetic neuropathy [12, 13]. 

The c.1117C>T (p.R373C) mutation comprises the only reported FBLN5 mutation linked to CMT; thus, the p.R373C position may represent a hotspot for mutations of FBLN5-related AD-CMT1 disease. Cellular studies that used fibroblast cells have demonstrated a loss of the recombinant forms of mutant fibulin-5 in the extracellular medium [14]. A normal structural density of basement membranes on Schwann cell surfaces is necessary for efficient myelination and axonal sorting [15]. The FBLN5 mutation may decrease myelination in the peripheral nerves. In addition, the loss of the FBLN5-integrin interaction augmented fibronectin signaling and drove integrin-induced ROS production [16, 17]. The excessive production of ROS may result in cellular toxicity for Schwann cells. 

In conclusion, this study demonstrated that FBLN5-related AD-CMT1 disease presented mainly as adult-onset motor-sensory neuropathy with hypertrophic features. 

## Acknowledgment 

We thank the participating family for their assistance. 

## Conflict of interest 

The authors declare no conflict of interest. 

**Table 1:** Motor and sensory nerve conduction findings in patients with AD-CMT neuropathy due to heterozygous c.1117C>T mutation in the FBLN5 gene.

Patient	I/1	II/2 (proband)	III/1
Motor nerve conduction			
Median nerve			
DML (ms)	6.15	7.83	5
MNCV (m/s)	28.8	23.1	31.5
CMAP amplitude (mV)	7.2	3.1	7.6
Ulnar nerve			
DML (ms)	4.15	6.21	NE
MNCV (m/s)	24.4	–	
CMAP amplitude (mV)	4.8	1.07	
Peroneal nerve			
DML (ms)	6.84	–	5.83
MNCV (m/s)	22.3	–	24.2
CMAP amplitude (mV)	2.6	–	5.5
Tibial nerve			
DML (ms)	7.33	–	NE
MNCV (m/s)	18.4	–	
CMAP amplitude (mV)	0.99	–	
Sensory nerve action potential amplitude (µV)			
Median nerve	8.1	–	9.6
Ulnar nerve	3.3	–	NE
Superficial peroneal nerve	–	–	14
Sural nerve	–	–	NE
F wave conduction velocities (m/s)	NE	–	32.4
H reflex latency (ms)	NE	–	44

DML = distal motor latency; MNCV = motor nerve conduction velocity; CMAP = compound muscle action potential; NE = not examined; – = no response.
